# The impact of educational interventions on modifying health practitioners’ attitudes and practice in treating people with borderline personality disorder: an integrative review

**DOI:** 10.1186/s13643-022-01960-1

**Published:** 2022-05-30

**Authors:** Pauline Klein, A. Kate Fairweather, Sharon Lawn

**Affiliations:** 1 Discipline of Public Health, College of Medicine and Public Health, Flinders University, Adelaide, South Australia 5001 Australia; 2 Discipline of Behavioural Health, College of Medicine and Public Health, Flinders University, Adelaide, South Australia 5001 Australia

**Keywords:** Borderline personality disorder, Structural stigma, Suicidality, Crisis care, Healthcare system, Health services, Community-based services, Integrative review, Evidence-based practice, Education and training

## Abstract

**Background:**

The rising prevalence of Borderline Personality Disorder (BPD) and suicidality represents substantial health burden worldwide. People with BPD experience high rates of crisis presentations and stigma when accessing health services. Educational interventions designed to modify health practitioners’ attitudes and practice in treating people with BPD may assist in addressing this stigma. The current review aimed to identify and explore existing educational interventions designed to modify health practitioners' attitudes and practice in BPD; and determine what impact educational interventions have on improving health practitioners’ responses towards people with BPD.

**Methods:**

A comprehensive search of the literature was undertaken in MEDLINE, CINAHL, PsycINFO, Scopus, Cochrane Library, and JBI Evidence-Based databases (from inception to February 2022). Secondary sources of literature included grey literature searches and handsearching the references of included studies as part of the comprehensive search strategy. The eligibility criteria included peer-reviewed empirical studies examining BPD-related educational interventions aimed at modifying health practitioners’ attitudes and practice in treating people with BPD. Quality appraisal of the included studies were completed using the Mixed Methods Appraisal Tool 2018 version (MMAT v.18) or the Joanna Briggs Institute (JBI) Checklist for Systematic Reviews and Research Syntheses Tool. Thematic Analysis informed data extraction, analysis, interpretation, and narrative synthesis of the data.

**Results:**

A total of nine papers containing 991 participants across a diverse range of studies including, quantitative, qualitative, mixed methods, and a systematic review were included in this integrative review. Several BPD-related educational interventions designed to modify health practitioners’ attitudes and practice in BPD exist. Findings suggest that training health practitioners in BPD-related educational interventions can enhance positive attitudes and change practice towards people with BPD; however, more high-quality studies are needed to confirm these conclusions.

**Conclusions:**

This review collated and summarized findings from studies examining the impact of BPD-related educational interventions on changing health practitioners’ attitudes and practice in treating this population. Results from this review may help inform future research, policy, and practice in stigma-reduction strategies which would improve the delivery of responsive health services and care for people with BPD.

**Systematic review registration:**

Open Science Framework (https://osf.io/7p6ez/)

**Supplementary Information:**

The online version contains supplementary material available at 10.1186/s13643-022-01960-1.

## Background

Recent calls for National Mental Health Reform underscore the growing concerns about mental health conditions [1–4] and suicide prevention [5, 6]; and the importance of delivering comprehensive approaches to improving healthcare systems’ capacity to meet the needs of vulnerable groups such as, people with BPD [7, 8]. BPD is a serious mental illness that is often complicated by problems with regulating emotions, interpersonal relationships, and suicidality [9]. Aside from the affects upon the person experiencing the condition, suicidality substantially impacts their carers/families and treating health practitioners [10]. Suicidality has been defined as suicide ideation (i.e., thoughts of suicide), self-harm (i.e., intentional non-suicidal injury), and suicide attempt (i.e., intentional directed injury) [11]. People with BPD are one of the most vulnerable populations affected by suicidality [12, 13]; and the rising prevalence of BPD [14–16] and suicidality [17] poses substantial health burden worldwide [15, 16, 18, 19].

Black et al. argued that suicidality may constitute the BPD symptom placing the greatest demand on healthcare systems [20]. People with BPD are often caught in a cycle of presenting to emergency departments in crisis [21] and remaining on long waiting lists for evidence-based therapies for BPD [22]. Studies estimating the global rates of health care utilization revealed that 10% of patients in outpatient services, and 15-22% of patients in inpatient services, were identified as having a BPD diagnosis [15–17, 20, 21]. A recent study investigating the rate of mental health presentations among people with personality disorders indicated that over the course of 3 years, 20.5% presented to emergency services; and 26.6% presented to inpatient services. In addition, people with personality disorders were twice as likely to access health services in crisis within 28 days of their last presentation, compared to people with other mental illnesses [23]. This repetitive help-seeking response to overwhelming distress and suicidality [13] has rendered BPD one of the most stigmatized mental illnesses in healthcare systems [12–14].

There are widespread reports of people with BPD experiencing discrimination and structural stigma in healthcare settings [12, 14, 22, 24, 25]. Structural stigma involves the organizational policies, cultural norms, and practices that inhibit health service access to particular group/s [26]. Structural factors, including the pervasive stigmatizing beliefs, attitudes, and practices to BPD in healthcare settings [26–29], play substantial roles in producing sub-optimal levels of health care for people with BPD [22, 24, 30]. Structural stigma also leads to major health inequities and outcomes [30, 31] in this population. The severity and nature of BPD, particularly in managing crisis presentations and the de-escalation of distress, can induce unconscious negative responses that is, countertransference, from health practitioners [32–34]. Mental health practitioners report finding people with BPD difficult to treat, manipulative, and treatment resistant [12]. Koehne et al.’s [13] study found that 89% of registered nurses working in mental health services (*N*=65) agreed with the statement that people with BPD are manipulative. Another study found that health practitioners viewed people with BPD who self-harm as attention seekers [35], rather than the behavior being a symptom consistent with the disorder. Suicidality has historically been judged harshly in healthcare systems [36], with some health practitioners finding it confronting to treat people who self-harmed or attempted suicide [37]. There are also reports of health practitioners denying treatment to people with BPD who present to health services in crisis [22]. These findings indicate the need for increased education and training in relation to BPD, to further assist health practitioners to support people with BPD who present to health services in crisis [30, 38].

There are currently limited studies on the effects of BPD-related educational interventions targeting health practitioners in healthcare settings, and whether these interventions are useful in changing health practitioners’ attitudes and practice in treating this population. The aims of this review were to: (1) identify and explore the existing evidence-base on educational interventions designed to modify health practitioners' attitudes and practice specific to BPD, including interventions that address BPD-related stigma in healthcare systems and, (2) determine what impact educational interventions have on improving health practitioners’ attitudes and practice in treating people with BPD.

## Methods

The integrative review was registered within the Open Science Framework (registration ID: https://osf.io/7p6ez/). A diverse range of study designs including quantitative, qualitative, mixed methods and systematic review, were included in the review. In accordance with integrative review methodology [39], this review sought to identify, synthesize, and summarize the current evidence-base on the impact of BPD-related educational interventions [40] on attitudes and practices to working with people with BPD among health practitioners from diverse disciplines, across various healthcare settings. Russell’s [41] five-stage integrative review process (problem formulation, literature search, data evaluation, data analysis, interpretation and presentation) guided the methodological rigor of this review.

### Literature search

The primary source of literature was identified via a search of electronic databases. The search strategy (see Additional file 1) was initially developed on PsycINFO in consultation with a research librarian prior to the search being applied to other electronic databases (from inception to February 2022): MEDLINE (Ovid), CINAHL (EBSCO Connect), Scopus (Elsevier), Cochrane Library (Wiley), and JBI Evidence-Based Database (Ovid). The secondary sources of literature included a search of grey literature including, Google search engine (Additional file 1), and handsearching the references of included studies to identify other relevant studies. Risk of selection bias was minimized by using these varied methods of study sourcing. The search included three categories of search key terms: (a) BPD; (b) Stigma; and, (c) Crisis care. The eligibility criteria (see Table 1) based on the Population-Concept-Context (PCC) framework [40] guided the study selection process during screening.

**Table 1:** Eligibility criteria

Population, Concept, Context [40] Criteria	
Population	Health practitioners including, psychiatrists, psychologists, social workers, mental health nurses, general practitioners, primary care nurses, and other mental health workers who treat people with BPD in healthcare settings such as, outpatients, inpatients, and community-based settings
Concept	Structural stigma specific to BPD and crisis care
Context	International peer-reviewed studies investigating educational interventions designed to modify health practitioners’ attitudes and practice in treating people with BPD in healthcare settings
**Inclusion criteria**	**Exclusion criteria**
Articles included were: Evaluated educational interventions designed to modify health practitioners’ attitudes and practice in treating people with BPD in an outpatient, inpatient, and community-based setting Evaluated structural stigma as an outcome in healthcare settings Original research including peer-reviewed publications on quantitative, qualitative, mixed-methods, and review designs Written in English language only	Articles excluded were: Evaluated health practitioners’ treating people with other mental illnesses Not reporting outcomes specific to borderline personality disorder and structural stigma Not conducted in non-clinical settings such as, educational institutions Studies of low quality

### Data evaluation

**Fig. 1:**
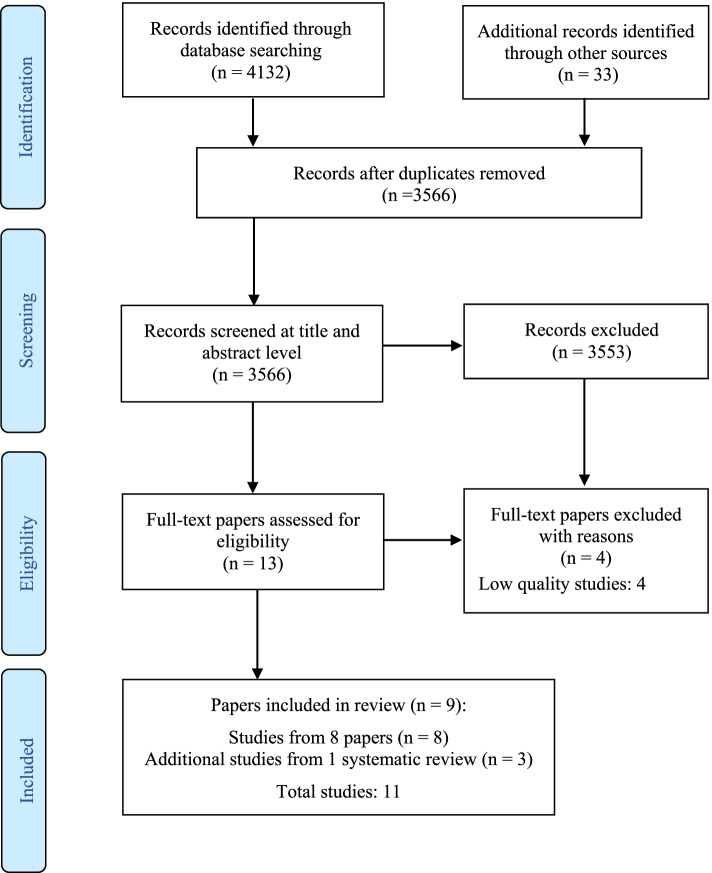
PRISMA flowchart of the selection of papers and studies for the integrative review

All citations identified from the search were uploaded into EndNote v.9 and Covidence, and duplicated articles were deleted by the lead author (PK). Citation screening and selection were undertaken using the Preferred Reporting Items for Systematic Reviews and Meta-Analyses (PRISMA) statement [42] (Additional file 2). Relevant citations were identified independently by two reviewers (PK and AKF) who screened the titles, abstracts, and full-text citations using eligibility criteria to ensure interrater reliability. Full-text citations of selected studies where then retrieved via Covidence and assessed against the inclusion criteria. Discrepancies in decisions regarding the inclusion of full-text citations were evaluated and resolved in Covidence by a third reviewer (SL) with clinical expertise in the field of mental health. Only empirical studies were included in this review. Data extraction (Table 2) was undertaken by the same reviewers (PK and AKF) following a meeting held by the research team (PK, AKF, SL) where the type of information to be extracted from those eligible studies was discussed and consensus reached. Initially, data extraction for all included articles was completed by the first author (PK). Subsequently, contents of the data extracted into the table were reviewed by the second author (AKF). Finally, any discrepancies between the two reviewers were to be resolved by the third author (SL); however, all concerns were resolved via consensus without involving the third reviewer. Results of searches are presented in the PRISMA Flow Diagram (Fig. 1).

**Table 2:** Data extraction of included studies on educational interventions for borderline personality disorder in healthcare

Author, Year, Country	MMAT v.18/JBI Quality Rating*	Population Type	Setting	Aim/Purpose	Study design/follow up	Intervention/Mode of delivery	Main Findings
Clarke et al. 2015 [43], UK	****	Multi-disciplinary teams	Inpatient setting	The aim of this study was to assess whether training in neurobiological underpinnings of Borderline Personality Disorder (BPD) could improve knowledge and attitude change within staff members working in a low secure inpatient setting	Within-subjects, quantitative questionnaire design / pre- and post- training and 2 month follow up	The Science of BPD	Attendance at the training session was associated with significant increases in theoretical knowledge, Perspective Taking and Locus of Origin scores. However, there were no changes observed in Empathic Concern scores. This research suggests that a relatively brief training session, that utilizes the neurobiological framework, can be effective in facilitating knowledge and attitudinal change for those working with BPD.
Commons Treloar et al. 2009 [44], Aus	***	Registered health practitioners	Aus / NZ health services	To examine two theoretical frameworks (cognitive-behavioral and psychoanalytic), and compare changes in clinicians’ attitudes towards deliberate self-harm behaviors in BPD	Exploratory Randomized Controlled Trial/ pre- and post-training and 6-months follow up	Cognitive Behavioral Therapy, Psychotherapy (both 45 min); Seminar discussions (45 min)	Compared with participants in the control group (*N*=22), participants in the cognitive-behavioral program (*N*=18) showed significant improvement in attitudes immediately after attending the program, as did participants in the psychoanalytic education program (*N*=25). The six-month follow-up revealed that only the psychoanalytic education group maintained significant changes in attitude. Results are discussed in terms of the use of relatively brief educational interventions in facilitating enduring attitude change toward working with this population.
Dickens et al. 2016 [35], UK	JBI, Level 1.b/ Level 4*	Mental health nurses		To collate the current evidence about interventions that have been devised to improve the responses of mental health nurses towards people with BPD	Systematic Review	Various interventions/duration of training	Eight studies were included in this review, half of which were judged to be methodologically weak, and the remaining four studies judged to be of moderate quality. Only one study employed a control group. The largest effect sizes were found for changes related to cognitive attitudes including knowledge; smaller effect sizes were found in relation to changes in affective outcomes. Self-reported behavioral change in the form of increased use of components of Dialectical Behavior Therapy (DBT) following training in this treatment was associated with moderate effect sizes. Mental health nurses hold the poorest attitudes to people with BPD.
Dickens et al. 2019 [45], UK	****	Mental Health staff	Inpatient / community settings	To evaluate/ explore mental health nurses’ responses to, and experience of, an educational intervention to improve attitudes towards people with a diagnosis of BPD	Mixed methods/pre- and post- training surveys and 4-month follow up/Focus Groups	Positive about Borderline including, Part 1: The Science of BPD (3-hr); and Part 2: Wot R U Like? Training (3-hr)/ Presentation, activities, discussion	Quantitative evaluation revealed sustained changes consistent with expected attitudinal gains in relation to the perceived treatment characteristics of this group, the perception of their suicidal tendencies and negative attitudes. Qualitative findings revealed hostility towards the underpinning biosocial model and positive appreciation for the involvement of an expert by experience. Conclusions: Sustained benefits of an educational intervention for people working with people diagnosed with BPD in some but not all areas. The study provides evidence for incorporation of a biosocial model into staff training and the benefits of expert by experience co-production. Mental health nurses believe that more well-resourced services are the key to improving care.
Keuroghlian et al. 2006 [46], USA	****	Mental Health clinicians including, psychiatrists, psychologists, counselors, nurses, and internists/primary care doctors	Medical Centers/Hospitals	Aims: (1) assess the effectiveness of Good Psychiatric Management workshops at improving clinicians’ attitudes to BPD and, (2) assess if changes in attitudes relate to the years of clinical experience	Before and after design/ pre- and post-training follow-up	Good Psychiatric Management Workshop (1-day)/ Didactic teaching; videos; case vignettes	Participants reported decreased inclination to avoid patients with borderline, dislike of patients with borderline, belief that BPD prognosis is hopeless, and increased feeling of professional competence, belief that they can make a positive difference and that effective psychotherapies exist. Less clinical experience was related to an increased feeling of competence. Findings demonstrate Good Psychiatric Management potential for training clinicians to meet population-wide needs related to BPD.
Knaak et al. 2015 [47], Canada	***	Health Practitioners including, social workers, psychiatrists, psychologists, counselors, nurses, students, director/managers	Health services	To identify whether a generalist or specialist approach is the better strategy for anti-stigma programming for disorders characterized by high levels of stigmatization; and to examine the extent an intervention led to change in perceptions towards people with BPD and mental illness.	Pre-post design/ pre- and post-training follow-up	BPD/ DBT Workshop (3-hr)	Although effectiveness cannot be conclusively demonstrated with the current research design, results are encouraging that the intervention was successful at improving healthcare provider attitudes and behavioral intentions towards persons with BPD. The results further suggest that anti-stigma interventions effective at combating stigma against a specific disorder may also have positive generalizable effects towards a broader set of mental illnesses.
Masland et al. 2018 [48], USA	****	Mental health clinicians, researchers, administrators	Medical Centre including outpatient, inpatient, residential, private practice.	To examine whether a 1-day training in Good Psychiatric Management can change clinician attitudes and beliefs and whether those changes persist over time	Repeated Measures Design/ pre- and post-training s and 6-month follow up	Good Psychiatric Management (1-day)/Lecture format; instructive case videos; case vignettes	Staff attitudes did not change immediately after training, but 6 months later had changed significantly. Findings indicate that brief training can foster enduring improvements in clinician attitudes and beliefs about BPD.
Pigot et al. 2019 [49], Aus	*****	Mental health clinicians, managers	Public mental health services	To understand the facilitators and barriers to real world implementation of a stepped care approach to treating personality disorders	Qualitative study	A Stepped Care approach/ post-training implementation at 18-mth follow-up	Participants identified personal attitudes, knowledge and skills as important for successful implementation. Existing positive attitudes and beliefs about treating people with a personality disorder contributed to the emergence of clinical champions. Training facilitated positive attitudes by justifying the psychological approach. Management support was found to bi-directionally effect implementation. Findings suggests specific organizational and individual factors may increase timely and efficient implementation of interventions for people with BPD.
Warrender 2015 [50], UK	*****	Nurses	Acute mental health services	To capture staff perceptions of the impact of health. Mentalization-based therapy skills (MBT-S) training on their practice when working with people BPD in acute mental health	Qualitative/Focus groups	MBT-S Training (2-day)/ Didactic teaching; role play; DVD clips	MBT-S Training promoted empathy and humane responses to self-harm, impacted on participants ability to tolerate risk and changed some perceptions of BPD. Staff felt empowered and more confident to work with people with BPD. The positive implication for practice was the ease in which the approach was adopted and participants perception of MBT-S as an empowering skill set which also contributed to attitudinal change.

Data extraction relevant to the review objectives and review question(s). *MMAT v.18 Quality rating: low = 1 to 2 stars; moderate = 3 stars; moderately high = 4 stars; high = 5 stars. *JBI Quality rating for level of evidence for effectiveness is level 1.b systematic review of RCTs and other study designs; and the level of meaningfulness is 4 - systematic reviews of expert opinion [51–53]

### Quality appraisal

To reduce the risk of bias, all articles included in the review were assessed for quality. The MMAT v.18 provides a structured checklist that was used to determine the methodology quality of the included quantitative, qualitative and mixed methods studies for inclusion into systematic reviews [51]. The JBI Checklist for Systematic Reviews and Research Syntheses tool was employed to appraise the methodological rigor of the systematic review paper that was included in this present integrative review [52, 53]. A series of meetings were initially held between the research team (PK, AKF, SL) to discuss the items within the quality appraisal tools and processes for assessing the methodological quality of the included studies. One reviewer (PK) conducted the quality appraisals in the first instance. Two reviewers (PK and AKF) then meet to review the quality appraisals of the studies and highlight any concerns; where issues were identified, resolution was achieved through discussion. A third reviewer (SL) was available to resolve any discrepancies between the two reviewers; however, all discrepancies were resolved without involving the third reviewer.

### Data analysis

Results of the review were analyzed and synthesized into a narrative summary relating to the study aims, research questions, and eligibility criteria (PCC). Data analyses involved: (1) quantitative data being summarized using descriptive statistics and frequencies [54] and, (2) thematic analysis of the qualitative data to organize, categorize, and interpret the key themes and patterns emerging from the data [55]. To ensure the trustworthiness and rigor of data abstraction and synthesis, a table was developed to capture the categories, codes, and summary of the key findings and interpretations regarding the impact of BPD-related educational interventions on modifying health practitioner attitudes and practice in treating people with BPD.

## Results

### Study characteristics

The search yielded 3,336 citations. Following screening of titles and abstracts, 13 relevant papers were retrieved in full-text and screened for eligibility. Of these papers, when critically appraising their quality, four [10, 28, 56, 57] were excluded because they were deemed to contain high risk (low quality) studies, resulting in a total of nine papers (*N*=9) being included in this review [35, 43–50]. Of these papers, eleven studies were selected for this review, including 3 additional studies from Dickens et al.’s [35] systematic review (see Fig. 1). Study characteristics, including MMAT v.18 or JBI quality ratings, sample, aim, study design, intervention, and main findings can be surveyed in Table 2. A summary of the key study characteristics is presented in Table 3.

**Table 3:** Summary of key study characteristics

	N	%
Participants	991	100
Study methodologies		
Quantitative studies	7	56
Qualitative studies	2	22
Mixed methods study	1	11
Systematic review	1	11
Healthcare setting		
Mental health services	5	45
Emergency departments	1	10
Hospital and health services	5	45
Occupation		
Medicine	159	16
Nursing	431	44
Allied Health	369	37
Education,	1	0.1
Administration	3	0.3
Director/Manager	8	0.8
Student	9	0.9
Other	11	1.1

### Methodological quality

Included studies were of moderate quality (*n*=3) [35, 44, 47], moderately high quality (*n*=4) [43, 45, 46, 48], and high quality (*n*=2) [49, 50]. Quality rating of studies based on the MMAT criteria [51] are provided in Table 2. In accordance with the MMAT, an overall score was not calculated for each quality criterion.

### Type of BPD-related educational interventions and outcomes

Findings revealed several existing BPD-related educational interventions designed to assist in changing health practitioners’ attitudes and practice in treating people with BPD [35, 43–50]. Most of the educational interventions were underpinned by theoretical and evidence-based psychological therapies known to be effective in the treatment of BPD, including Dialectical Behavior Therapy (DBT) [47], Cognitive Behavioral Therapy (CBT), Psychoanalytic Therapy [44], and Mentalization-based Therapy Skills (MBT-S) Training [50]. Other studies developed, designed, and evaluated new BPD training program/s as part of their research study [35, 45]. All studies included stigma outcome measures such as, cognitive (e.g., beliefs about etiology, knowledge of BPD), affective (e.g., attitudes, desire for social distance), and/or behavioral outcomes (e.g., intent to practice, improved clinical skills). While all studies measured attitudinal change among health practitioners following participation in BPD-related education [35, 43–50]; other studies also measured health practitioners’ clinical practice, organizational change [35, 49], and patient outcomes [35]. Additional file 3 presents the BPD-related educational interventions, the structural factors that the intervention intended to modify, and the stigma-reduction outcomes of the included studies.

### Type of quantitative evaluation measurements

All studies evaluated at least one educational intervention, with all but two studies [49, 50] undertaking quantitative pre- and post-surveys to investigate attitudinal/stigma-related measures. Most of these studies used validated and reliable attitudes/stigma assessment tools that had been tested within BPD populations [43, 44, 47, 48] such as, the Perspective Taking Scale [58], the Attitudes Towards Deliberate Self-Harm Questionnaire (ATDSHQ) [59], and the Opening Minds Scale for Healthcare Providers (OMS-HC) [60]. The validity and reliability of measurement tools employed in other studies were deemed questionable [35, 45], or not psychometrically tested [43], so those findings need to be interpreted with caution [35].

### Study findings

#### Impact of BPD-related educational interventions on stigma-reduction outcomes

Overall, findings of this review suggests that educational interventions for BPD may positively impact health practitioners’ attitudes [35, 43–50] and practice [35, 49], in treating people with BPD presenting to health services in crisis. Commons Treloar et al.’s [44] Randomized Controlled Trial (RCT) compared the effect of two educational interventions (i.e., CBT and Psychoanalytic Therapy) on changing health practitioners’ attitudes to treating people with BPD and understanding Deliberate Self-Harm (DSH). Compared to the control group, findings indicated that both the CBT and Psychoanalytic Therapy educational interventions effected statistically significant changes in participants attitudes immediately following training with medium effect sizes. Psychoanalytical Therapy was also found to be effective in sustaining participants’ attitudinal changes at six-month follow-up with a small effect size. Knaak et al. [47] study found that DBT training improved health practitioners’ attitudes to both BPD and mental illness more broadly. Greater attitudinal improvements were reported among the health practitioners in the BPD group, compared to the mental illness group. Overall, a significant decrease in participants’ stigma-related scores were evident on: ‘disclosure/help-seeking behaviors of people with BPD’; ‘intent-to-practice skills in treating people with BPD’; and ‘preferences for social distance’ items on the OMS-HC scale. Herschell et al. [35] detected improvements in participants’ attitudes to BPD which were sustained for up to two years post training in DBT.

In contrast, Pigot et al. [49] and Stringer et al.’s [35] participants self-reported no change in attitudes to treating people with BPD in response to the training - despite reporting changes in outcomes associated with clinical practice [49] and patient outcomes [35]. Pigot et al.’s [49] study found that the training alone was not sufficient in changing health practitioners’ practice, but rather the combination of the training and practical experience of working with people with BPD in their health service/s, helped to build staffs’ competence and confidence in treating this population. Similarly, Herschell et al’s [35] participants reported that their skills in working with people with BPD in their health service had improved following training in DBT. Stringer et al’s [35] study trained nurses in a collaborative care program and found significant improvements in patients’ BPD-related symptoms.

Studies investigating stigma-related outcomes specific to affective (e.g., attitudes), cognitive (e.g., knowledge), and/or behavioral (e.g., practice) outcomes pre- and post-training [35] reported positive changes among participants on the following items, for example: feeling they can ‘make a positive difference in the lives of patients with BPD’ [46]; being ‘willing to disclose the BPD diagnosis to patients’ [48, 54]; and amelioration in ‘preferences for avoiding care of a BPD patient’ [46]. However, Dickens et al.’s [35] review suggests that education targeting affective or cognitive adjustments may be insufficient to change more embedded structural inequities in healthcare systems. See Additional file 3 for further details on the impact of BPD-related educational interventions on structural stigma-reduction outcomes of included studies.

Components of BPD-related brief educational interventions that participants reported as useful were the experiential aspect of including expert by experience (i.e., people with BPD) personal testimonies (both face-to-face and videos) [49], understanding empathy within the context of the therapeutic practitioner-client relationship, and training in developing health practitioners’ skill sets (i.e., a toolkit of easy-to-use-strategies) for working with people with BPD [49, 50]. Learning about introductory therapeutic approaches and the etiology of BPD were viewed by some participants as less useful components of BPD-related educational intervention/s [50].

##### Synthesis of the findings identified several emerging themes below

**Fig. 2:**
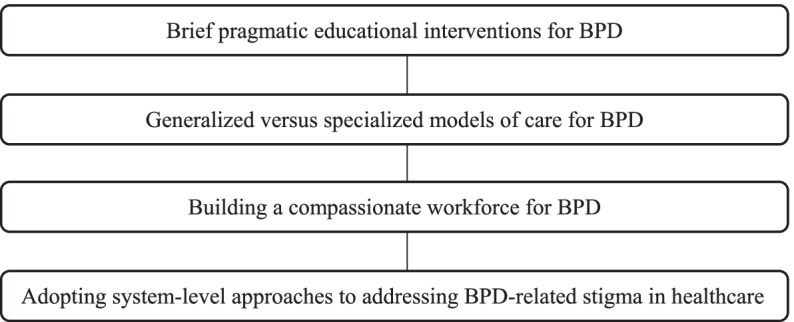
Findings on the key themes of the integrative review

These related to the key facilitators, challenges, and barriers in the design and delivery of BPD-related educational interventions for health practitioners who treat people with BPD. Macro- and micro-level factors, potentially contributing to the BPD-related structural stigma in healthcare systems are considered within a broader stigma-reduction approach to tackling this important public health problem. Figure 2 presents the key themes that emerged from the data.

#### Brief pragmatic educational interventions for BPD

Studies in the present review identified several factors as key in the design and delivery of BPD-related educational interventions. Several studies (*n*=5) highlighted the usefulness of training health practitioners in brief pragmatic BPD-related educational interventions that were underpinned by the realities and constraints of clinical demand and service provision, as well as relevant to the health setting in which care was being delivered [45–48, 50]. Training busy health practitioners using brief educational sessions were found to support changes in staff attitudes and practice in BPD [46, 47, 50]. In addition, Dickens et al.’s [45] study found that participants from a community day hospital viewed the educational intervention as relevant to their work environment because they had the time to engage with patients and build therapeutic relationships. In contrast, participants working in inpatient wards found that the training was not conducive to their work environment. Further, they expressed frustration about receiving education on therapeutic approaches when time, resources, ongoing staffing issues, and clinical demand presented real barriers to implementation within an acute health setting. Participants also reported no change in their beliefs regarding ‘people with BPD being time-consuming’ and raised concerns regarding the challenges of allocating extra time to build relationships with people with BPD. This review highlighted the importance of educational interventions being customized to various health settings/context to help health practitioners to support people with BPD, and ultimately enhance patient outcomes [49]. Dickens et al.’s [45] investigation highlighted that combining work teams from diverse settings (i.e., staff from a community day hospital service and acute inpatient wards), into the same training session divided the teams when the course content did not match the realities of the work practices within these vastly disparate settings. As one participant stated, “it kind of split the whole room apart because it (the content) was very much, ‘this is how you're meant to do it’...‘you're doing it wrong.’” (p.2618).

Dickens et al.’s [45] study also discussed co-designing educational interventions with experts by experience (in this case people with BPD) as an innovative approach to improving the delivery of health services for BPD. Participants commented that the inclusion of people with BPD in the education and training programs was useful because it helped them to gain an intimate understanding of the lived experiences of those with BPD and their emotional distress and recovery. The educational programs appeared to positively change practitioners’ attitudes towards treating people with BPD. Participants also indicated that learning about people with BPD lived experiences could assist them to offer hope to other patients with similar problems. In addition, participants reported that they valued people with BPD recommendations on how to improve practice as the suggestions were based on personal experiences and the realities of limited services and resources. Moreover, participants valued the discussions held with the patient’s mental health practitioner regarding her treatment and progress. Overall, findings from this review suggests that health practitioners find BPD-related brief educational interventions useful in assisting them in transitioning the learnings from the training into practice [45, 49, 50].

#### Generalized versus specialized models of care for BPD

This theme explores the concept of tailoring specialized therapeutic approaches that have been found to be effective in the treatment of BPD into brief educational interventions for use in generic health services. Warrender [50] found that a specialized therapeutic approach (i.e., Mentalization-based Therapy (MBT)) that had been tailored into a brief educational intervention (i.e., MBT-S) was perceived by nursing staff as an acceptable and useful approach in an acute mental health service context. Staff reported that the brief MBT-S approach gave them a theoretical perspective for understanding BPD and provided them with a skill set of easy-to-use strategies to implement when working with people with BPD. Training health practitioners in generalized brief intervention approaches may also address the challenges in the delivery of BPD-related services in generic settings. For instance, some participants held conflicting views about the role of health practitioners in treating BPD and presented several barriers to delivering specialized therapeutic approaches in their health service [45, 49]. As one participant in Dicken et al.’s [45] study stated: “Were not therapists, we’re psychiatric nurses and if … this is a specific therapy this person needs … we can’t deliver that … ” (p. 2619). These findings suggest that some health practitioners may consider that the skills needed to effectively engage people with BPD are outside of their professional role/capabilities. Despite some health practitioners’ reluctance to deliver specific interventions, the overall findings of this review suggest that delivering brief interventions is useful in assisting health practitioners treating people with BPD in generic health services [45, 50].

#### Building a compassionate workforce for BPD

This theme centers around building a compassionate workforce that is inclusive of BPD service provision. This involves embedding practices that are compassionate, empathetic, and caring [50], as well as responsive to the specific needs of people with BPD who present to health services in crisis [49]. Of the studies that incorporated empathetic approaches [35, 43, 44, 48, 50], all but one [45] identified that training in BPD-related interventions positively modified health practitioners’ attitudes and practice, and facilitated the delivery of person-centered approaches to people with BPD. Commons Treloar et al. [44] observed that educating health practitioners in a Psychoanalytic approach to understanding the unconscious processes underlying BPD and DSH, was more effective at eliciting empathetic responses from participants, than was education in the conceptualization of the emotional/behavioral disturbances associated with BPD and DSH (as part of the CBT approach). Warrender [50] trained health practitioners to understand empathy as the foundation for building therapeutic alliances with patients. Participants in this study subsequently reported feeling less frustrated and that their working relationships with people with BPD and DSH had improved. Another study [43] trained health practitioners in understanding the biosocial underpinnings of BPD, and found significant increases in health practitioners ‘perspective taking’ (i.e., an aspect of empathy). However, no significant differences were found in health practitioners ‘empathic concern’ for people with BPD following training. Pigot et al.’s [49] participants reported experiencing increased compassion towards people with personality disorders following training, which encouraged their continued engagement in the change process that was occurring within their health service. In contrast, some participants were much more resistant to change and expressed pessimistic views about offering empathy to people with BPD, despite their involvement in the training [45]. Overall, review findings supported the need for ongoing training in BPD-related approaches to further upskill health practitioners in the delivery of compassionate health services and care for people with BPD [35, 43–45, 49, 50].

#### Adopting system-level approaches to addressing BPD-related stigma in healthcare

**Fig. 3:**
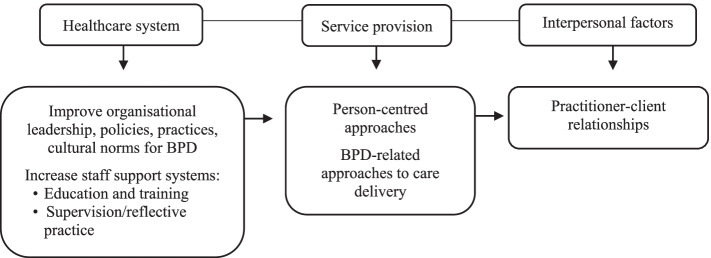
A systems approach to responsive service provision for BPD

This theme concerns the need for a system-wide approach to addressing institutions organizational leadership, policies, cultural norms, and practices contributing to BPD-related structural stigma in healthcare systems (See Fig. 3). Several structural problems were identified as particularly pertinent to improving BPD-related service provision, education, and training. These included the need to: deliver more responsive, person-centered approaches to BPD-related health services [35, 49, 50]; give health practitioners more autonomy and self-management over client contact to enable time to build practitioner-client relationships with people with BPD, particularly staff in emergency departments and on acute hospital wards [35]; and increase health practitioners understanding that the treatment and management of BPD is ‘core business’, across *all* health practitioners/services [45, 49]. Pigot and colleagues [49] recognized several themes pertaining to specific organizational and individual factors that can also help to facilitate system-level change in healthcare systems. These included: providing access to ongoing education and training, and supervision in BPD; the need for clinical champions, management support, governance to minimize client risk, and change management processes/plans to support implementation of BPD-related interventions in healthcare settings. It was also noted that leadership, governance, and managerial support for the change process was instrumental in the successful integration of the intervention into health service practices. Overall, review findings highlight that greater investment in system-level approaches are needed to improve health systems’ responses to BPD-related service provision.

## Discussion

This integrative review systematically identified, organized, and synthesized a narrative summary of the international evidence-base on BPD-related educational interventions targeting multi-disciplinary health practitioners’ attitudes and practice in treating people with BPD across various healthcare settings. As a whole, results of this review suggests that BPD-related educational interventions have considerable potential to positively impact health practitioners’ attitudes [35, 43–48, 50] and practice [35, 49], while mitigating the effects of structural stigma on the provision of health services and care for this population [48]. However, while affective, cognitive, and behavioral changes in response to training health practitioners in BPD-related educational interventions where evident in some studies [35, 46]; education targeting affective or cognitive adjustments alone may not be sufficient to modify the structural inequities that currently exist within healthcare systems [35]. In addition, it appears that health practitioners possessed substantially higher stigma scores towards people with BPD, relative to the levels of stigma related to people with mental illness more broadly [47]. This confirms previous evidence indicative of BPD being a highly stigmatized disorder in healthcare settings [26, 36], where the severity of stigma appears to vary depending on the type of disorder [47, 61]. These findings strengthen the call for disorder-specific stigma-reduction approaches to better support health practitioners to appropriately respond to the specific BPD-related symptoms experienced by people presenting to health services in crisis [47].

Consistent with Dickens and colleagues’ [35] review, the present review finds too few high-quality studies to confirm whether existing BPD-related education and training can, in fact, assist change in health practitioners’ responses to treating people with BPD. While all studies included in this review examined the impact of educational interventions on modifying health practitioners’ attitudes to treating people with BPD, the effect sizes reported were only small to moderate, at best. Three effectiveness studies undertaken to assess health practitioners’ attitudes and practices in treating people with BPD in healthcare settings, yielded statistically significant changes in participants’ responses to working with people with BPD. One of these studies was deemed to be of moderate methodological quality [44]. The other two studies (in Dickens et al.’s review) [35] claimed to be RCTs, but had no control groups, and thus, were excluded from this review based on low methodological quality. The quality of the included studies was moderate to moderately high [35, 43–49], or low quality (*n*=4) [35]. The response rates were relatively high (approximately 60%) [62] at post-training and/or follow-up. Two studies [35, 48] had low response rates (27% or less) [62] for follow-up surveys. The two qualitative studies included in this review [49, 50] were rated as high quality, and while they provided an in-depth knowledge and understanding of health practitioners’ perspectives and lived experiences of their participation in the educational interventions, these findings are constrained due to lacking generalizability to the broader BPD population [54]. Only two studies investigated modifications in clinical practice [35, 49] but the evidence was based on self-reported outcome measures. Few studies measured organizational change (*n*=2) [35, 49], and patient outcomes (*n*=1) [35] in response to health practitioners’ participation in the training. Consequently, more high-quality studies are recommended to determine the effectiveness of BPD-related educational interventions and confirm current evidence.

Several key themes emerged from the data based on the current BPD-related educational interventions targeting health practitioners’ attitudes and practice. Themes encompass: training health practitioners in brief pragmatic educational interventions for BPD; the design and delivery of generalized versus specialized models of care for BPD; building a compassionate workforce for BPD; and, adopting system-level approaches to addressing BPD-related stigma in healthcare. Present findings suggest that embedding brief BPD-related educational interventions within the context of real-world healthcare settings can improve the uptake and delivery of training approaches in health services [45, 47, 49, 50]. This includes providing health practitioners with brief, time-efficient educational sessions in evidence-based approaches for BPD. Further, by tailoring brief educational interventions to the needs of people with BPD, health practitioners [49], and the clinical setting in which the intervention was being delivered, there was greater success in affecting positive change in health practitioners’ attitudes and practice [45, 49, 50], compared to educational interventions that were not viewed as relevant to health practitioners or the setting [45]. Implementation of BPD-related interventions was realized where management styles considered staff needs [49], and elicited positive change in staff attitudes [48, 63]. These factors need to be considered within a system-wide approach to leading change in the provision of health services for BPD [6, 49].

This study made tacitly clear that the importance of understanding an organization’s culture is vital, as was understanding how cultural norms and practices in health settings can impede the successful implementation of service-level changes [49, 64]. Studies [45, 49] comparing health practitioners’ attitudes and practice in the implementation of a BPD intervention in various health sites found substantial differences in the uptake and delivery of the intervention across the sites. For instance, participants who perceived that people with BPD required specialized therapeutic treatment approaches were more reluctant to deliver the intervention within their service, than the health practitioners in other health sites where change processes for BPD-related practices were embraced [45, 49]. This reflects differences in the group dynamics of health practitioners across various health sites. According to Social Identity Theory [65] (i.e., the theory of group membership and its influence on individual members’ attitudes and behavior), the social identities of groups are important drivers of learning and performance within the workplace; and thus, approaching specific groups with defined identities/roles is key to affecting attitudinal and behavioral change. Therefore, educational interventions aimed at changing organizational culture need to consider identity-based group dynamics, along with other organizational and individual level factors, to effectively drive change [66]. One context where there is a pressing need for delivery of effective BPD-related brief interventions [45, 50] is in generic health services, particularly in emergency departments or acute hospital settings with high prevalence of health service utilization among people with BPD [14–16, 18].

Another key theme in this review was the clear need for healthcare systems to build a compassionate workforce [4, 7] that is inclusive of BPD-related health service provision and meets the needs of people with BPD. The emergence of this theme is consistent with recent government recommendations for improving the standards of care of people with mental illness through the timely provision of compassionate, empathetic, and responsive health services [1, 2]. Our review identified several existing educational interventions that were founded on evidence-based therapeutic approaches for treating BPD, some of which were useful in supporting health practitioners to elicit compassionate and empathetic responses to people with BPD experiencing distress, as well as reducing BPD-related stigma [44, 46, 48–50].

Interestingly, Common Treloar et al.’s [44] study demonstrated that training health practitioners in a Psychoanalytic approach was more effective in eliciting empathetic responses from health practitioners, than was a CBT approach. These findings are indicative of the distinct differences between the theoretical underpinnings of Psychoanalytic Therapy and CBT. Training in the CBT approach entailed understanding DSH as a coping mechanism for regulating strong emotional states which seemed to imply that a level of consciousness was involved, which in turn, resulted in health practitioners feeling less empathetic towards people with BPD who repeatedly presented to health services following DSH. Alternatively, learning about the unconscious processes of Psychoanalytic approaches seemed to assist health practitioners to recognize and consolidate their understanding of BPD as a complex condition, and elicited greater empathy towards people with BPD. It appears that health practitioners are more likely to feel empathy towards a person who is considered not responsible, on a conscious level, for their behavior. Results from a recent review [24] suggest that increasing health practitioners’ understanding of the complexities of BPD and its associated symptoms/stigma can enhance health practitioners’ empathy towards BPD as well as address structural stigma associated with this disorder in healthcare systems.

The final theme emphasizes the need to incorporate education and training within a comprehensive system-level, multi-strategy approach [2–4, 7], to better address how BPD-related health services are delivered in healthcare settings. Results from this review suggest that education and training as a stand-alone strategy is unlikely to be sufficient to instill adequate and sustained changes in health practitioners’ attitudes and practice in BPD. Rather, a system-wide approach to tackling structural stigma at both the macro- and micro-levels of institutions is required, including implementing coordinated and targeted changes to organizational leadership approaches, policies, culture, and practices [5, 18, 49].

There are several limitations to this review. The lack of studies possessing high methodological quality, including RCTs, limits this review from clearly establishing causality or ascertaining whether training health practitioners in BPD-related interventions have had an impact on modifying staff responses in treating people with BPD. In addition, while other studies on BPD-related educational interventions targeting health practitioners’ attitudes or practice in BPD may exist, the rigor maintained by this review required their exclusion based on the eligibility criteria, which included studies not freely accessible. Future research is needed to identify whether changes in health practitioners’ attitudes transform into changes in organizational culture, practice, and patient outcomes such as, improved practitioner-client relationships, patient satisfaction, and reduced rates of suicidality. Further studies investigating the effectiveness of individual components of educational interventions are also needed to determine which aspects of the training specifically facilitate mechanisms for affecting positive change in health practitioners’ attitudes and practice in BPD [45, 67].

## Conclusion

Review of existing BPD-related educational interventions yielded modest effects regarding whether providing training in BPD is sufficient in assisting health practitioners to positively change their responses to this population. It is evident from the studies reviewed, that multi-level, multi-strategy system-wide approaches [2–4, 7] are needed to upskill health practitioners in the effective treatment of BPD, and embed these interventions into health service provision [18]. However, more high-quality studies are needed to confirm this [35]. Overall, it is anticipated that the outcome of this review will inform future research, policy, and practice relating to stigma-reduction strategies that, once adopted, can improve the delivery of responsive health services and care for people with BPD [57].

## Supplementary Information

**Additional file 1.** Search strategy for various databases.

**Additional file 2.** PRISMA 2009 Checklist.

**Additional file 3.** Impact of BPD-related educational interventions on structural stigma-reduction outcomes.

## Data Availability

Not applicable.

## Abbreviations

BPD: Borderline personality disorder
CINAHL: Cumulative Index to Nursing and Allied Health Literature
JBI: Joanna Briggs Institute
MMAT: Mixed Methods Appraisal Tool 2018 version
PCC: Population-Concept-Context framework
PRISMA: Preferred Reporting Items for Systematic Reviews and Meta-Analyses
RCT: Randomized Controlled Trial
DBT: Dialectical behavior Therapy
CBT: Cognitive Behavioral Therapy
MBT: Mentalization-based Therapy
MBT-S: Mentalization-based Therapy Skills
ATDSHQ: Attitudes Towards Deliberate Self-Harm Questionnaire
OMS-HC: Opening Minds Scale for Healthcare Providers
DSH: Deliberate Self-Harm
