# Supplementary material for: The impact of educational interventions on modifying health practitioners’ attitudes and practice in treating people with borderline personality disorder: an integrative review
Source: Syst Rev. 2022 May 30;11:108. doi: 10.1186/s13643-022-01960-1 (PMC9150362; doi:10.1186/s13643-022-01960-1)
Supplement: Supplementary file 1 — Additional file 1. Search strategy for various databases. [file 13643_2022_1960_MOESM1_ESM.docx]

**Additional file 1.** Search strategy for various databases

| **Search**  **No.** | **Searches** |
| --- | --- |
| 1 | Borderline Personality Disorder/ |
| 2 | Personality Disorders/ |
| 3 | (borderline adj4 (person* or client* or patient* or consumer* or carer* or famil*)).tw,id. |
| 4 | (BPD or BPDs).tw,id. |
| 5 | (personality adj4 disorder*).tw,id. |
| 6 | (emotional* unstable adj4 (person* or client* or patient* or PD or PDs or state* or disorder*)).tw,id. |
| 7 | or/1-6 |
| 8 | crisis intervention/ or suicide prevention/ or risk/ |
| 9 | crisis intervention services/ or emergency services/ or health services/ or mental health services/ or mental health care/ or primary health care/ |
| 10 | suicid*/ or self-destructive behav*/ or attempted suicide/ or suicidology/ or self-injurious behav*/ or suicidal ideation/ or self-mutilat*/ |
| 11 | (cris* or acute or emergenc* or critical).tw,id. |
| 12 | (help-seeking or help seeking behav*).tw,id. |
| 13 | (suicid* or self-harm or self-injur* or (self adj3 (harm or injur*))).tw,id. |
| 14 | or/8-13 |
| 15 | stereotyping/ or stigma*/ or prejudice/ or discrimination/ or marginali*/ or exp attitude/ |
| 16 | ((negative or positive) adj3 attitude*).tw,id. |
| 17 | (discriminat* or marginali* or reject* or exclu* or stigma*).tw,id. |
| 18 | (health services adj4 (experienc* or perspective* or perception* or view*)).tw,id. |
| 19 | (refus* or den*) adj3 (service* or treat*).tw,id. |
| 20 | ((anti-stigma or anti stigma or stigma-change or stigma reduction) adj3 (intervention* or program* or strategy* or education* or awareness)).tw,id. |
| 21 | or/15-20 |
| 22 | 7 and 14 and 21 |
| 1. 23 | limit 22 to english language |

Grey literature search strategy

Key words:

“Borderline personality disorder” “BPD” “consumer” “carer” “family” “clinician” “healthcare provider” “health practitioner” “crisis intervention” “crisis care” “suicide prevention” “help-seeking” “suicide” “suicidal behaviour” “self-harm” “stigma” “discrimination” “attitude” “refuse service” “refuse treatment” “anti stigma intervention” “stigma reduction intervention” “anti stigma program” “stigma reduction program” “anti stigma education” “anti stigma awareness”.
