# Supplementary material for: The impact of educational interventions on modifying health practitioners’ attitudes and practice in treating people with borderline personality disorder: an integrative review
Source: Syst Rev. 2022 May 30;11:108. doi: 10.1186/s13643-022-01960-1 (PMC9150362; doi:10.1186/s13643-022-01960-1)
Supplement: Supplementary file 3 — Additional file 3. Impact of BPD-related educational interventions on structural stigma-reduction outcomes. [file 13643_2022_1960_MOESM3_ESM.docx]

**Additional file 3.** Impact of BPD-related educational interventions on structural stigma-reduction outcomes

| **Educational interventions** | **Structural factors aimed to modify** | **Stigma-reduction outcomes** |
| --- | --- | --- |
| The Science of Borderline Personality Disorder (Neuro-biological) (Clarke et al. 2015) | Attitudes, knowledge, empathy | * Sustained positive changes in:   - increased theoretical knowledge on genetics, neuroanatomy, and cognitive dysfunction - increased ‘perspective taking’ scores - increased ‘locus of origin’ scores. |
| Cognitive-behavioral therapy education program  Psychoanalytic education program  (Commons Treloar et al. 2009) | Attitudes  Knowledge, attitudes | *Short-term positive attitude changes to borderline personality disorder/deliberate self-harm  ***Sustained positive attitudes to borderline personality disorder/deliberate self-harm.. |
| Positive About Borderline  The Science of Borderline Personality Disorder (Biosocial)  Wot R U Like? (Expert by experience) (Dickens et al. 2019) |  | **Sustained cognitive/emotional attitudinal changes in:   - perception of treatment characteristics - perception of suicidal tendencies - stigma-related attitudes. |
| DBT Program (Herschell et al. 2014) | Attitudes, beliefs, intent to practice, confidence | Short-term positive changes in:   - attitudes towards BPD - Belief/confidence about the effectiveness of DBT - Perceived use of DBT. |
| Good Psychiatric Management workshop (Keuroghlian et al. 2006) | Attitudes, beliefs, confidence | Short-term positive changes in:   - willingness to treat - readiness/competence to treat - belief that symptoms cause distress/low-self esteem - ability to make a difference - belief of effective treatment/recovery - reduced dislike/avoidance of patients - interest in receiving more training. |
| Dialectical Behavior Therapy brief intervention (Psychoeducation) (Knaak et al. 2015) | Attitudes | Short-term positive attitude changes in:   - perceptions of borderline personality disorder and mental illness in general - Disclosure/help-seeking   ***Sustained positive attitude changes:   - reduced preferences for social distance. |
| Good Psychiatric Management workshop (Masland et al. 2018) | Attitudes, belief, competence, confidence | Short-term changes in:   - ability to make a difference - effective treatments   ***Sustained positive changes in:   - reduced dislike of patients - willingness/competence to treat - willingness to disclose diagnosis - knowledge of distress/low self-esteem - increased empathy - belief in change/recovery. |
| Self-instructional Program for BPD  (Miller and Davenport 1996) | Knowledge, attitudes, practice | Short-term changes in:   - Knowledge of BPD - attitudes towards BPD - intent to treat people with BPD. |
| A Stepped Care Intervention for Personality Disorders (Pigot et al. 2019) | Knowledge, attitudes, confidence, practice, organizational values, culture change | ****Long-term sustained changes in:   - attitudes towards the psychotherapeutic approach - knowledge of effective therapies potentially reduced stigma - combination of training and practice increased confidence to treat - organizational changes - intervention embedded into core practice in one mental health services site - identified need for ongoing training to assist the implementation of the intervention. |
| Systems Training for Emotional Predictability and Problem Solving (STEPPS) Program (Stringer et al. 2015) | Knowledge, attitudes | Short-term changes in:   - knowledge of BPD - attitudes towards BPD. |
| Mentalization-based Therapy Skills training  (Warrender 2015) | Knowledge, attitudes, practice | Short-term knowledge/attitude changes in:   - treating people with BPD - increased understanding of empathy, self-harm, therapeutic relationships - reduced stigma-related attitudes   Short-term changes in practice:   - capacity to tolerate risk involving self-harm/suicidality - demonstrated increased empathy and appropriate responses to self-harm/suicidality - reduced stigma-related behavior to BPD - engagement in supervision. |

*Short-term refers to changes in attitudes/practice immediately following training, **Sustained refers to changes in attitudes/practice at 2- and 4-month follow-up, ***Sustained refers to longer-term changes in attitudes/practice at 6-month follow-up, ****Long-term sustained changes in attitudes/practice at 18-month follow-up.
